# A Statistical Assessment of the Impact of Agricultural Land Use Intensity on Regional Surface Water Quality at Multiple Scales

**DOI:** 10.3390/ijerph9114170

**Published:** 2012-11-15

**Authors:** Weiwei Zhang, Hong Li, Danfeng Sun, Liandi Zhou

**Affiliations:** 1 Institute of Agricultural Integrated Development, Beijing Academy of Agriculture and Forestry Sciences, No.9 Shu Guang Hua Yuan Middle Road, Beijing 100097, China; Email: zhangwei492@163.com (W.Z.); liandizhou@126.com (L.Z.); 2 College of Natural Resources and Environmental Science, China Agricultural University, Beijing 100094, China; Email: sundf@cau.edu.cn

**Keywords:** agricultural land use intensity, water quality, multi-scale, linear regression

## Abstract

Understanding the effects of intensive agricultural land use activities on water resources is essential for natural resource management and environmental improvement. In this paper, multi-scale nested watersheds were delineated and the relationships between two representative water quality indexes and agricultural land use intensity were assessed and quantified for the year 2000 using multi-scale regression analysis. The results show that the log-transformed nitrate-nitrogen (NO_3_-N) index exhibited a relationship with chemical fertilizer input intensity and several natural factors, including soil loss, rainfall and sunlight at the first order watershed scale, while permanganate index (COD_Mn_) had a positive relationship with another two input intensities of pesticides and agricultural plastic mulch and organic manure at the fifth order watershed scale. The first order watershed and the fifth order watershed were considered as the watershed adaptive response units for NO_3_-N and COD_Mn_, respectively. The adjustment of agricultural input and its intensity may be carried out inside the individual watershed adaptive response unit. The multiple linear regression model demonstrated the cause-and-effect relationship between agricultural land use intensity and stream water quality at multiple scales, which is an important factor for the maintenance of stream water quality.

## 1. Introduction

Land use intensity is one of the most significant forms of land cover modification, and can have a major detrimental impact on terrestrial and aquatic ecosystems [1,2], and also directly influence human and ecosystem health. Many developed countries are experiencing environmental pollution due to intensive agricultural activities, including intensive crop and livestock production [3]. This is also the true for the fast developing countries, such as China. From a land use perspective, intensive agricultural activities have been identified as the major sources of non-point source pollutants and are known to alter and impact the quality of the receiving water bodies. As an environmental factors that relate directly to human health, water quality is always subject to degradation when agricultural land use intensity is too high [4]. Thus, understanding the effects of intensive agricultural land use activities on water resources is essential for natural resource management and environmental improvement. However, these effects on water quality conditions are difficult to determine because of the complex relationships between agricultural land use activities and water quality.

In previous studies water quality was generally linked to land use inside the catchments area. Several studies have found that land use has a strong influence on the receiving water body quality [5,6,7]. The majority of studies about the effects of land use on water quality have focused on either deterministic modeling, or spatial, or statistical analyses. Examples of modeling studies include those performed by Tong and Chen [5], Chaplot *et al.* [8], Cao *et al.* [9], Bhattarai *et al.* [10], *etc*. which have adopted the existing watershed-scale hydrological variables and nonpoint-source pollution models to evaluate or predict how land use/land cover scenarios affect water quality. Since modeling methods need long-term water quality monitoring data and regional parameters are difficult to obtain, current modeling methods are still developmental and confined to mechanism studies in local watersheds. Consequently, there are more studies that have adopted statistical methods such as correlation analysis [11,12], single linear regression analysis [13,14], multiple linear regression analysis [15,16,17], nonparametric statistical analysis techniques [18], *etc.* to examine the relationships between watershed land use/land cover and water quality.

Since no statistical significant relationships between land uses and nitrate level were found when using the whole basins, contributing areas inside buffer zones were developed by Basnyat *et al* [19]. There have been more subsequent studies taking buffer zones as analysis units to explore water quality characteristics and their relationships [20,21,22]. The definition of contributing zone may open additional ways of visualizing the problem. The previous studies have demonstrated that the contributing zone is influenced by many factors, including the water-quality parameter being assessed and geomorphic/climatic setting of the watershed [19]. To some extent, buffer zones with multi-scale characteristics, created using the distance from the stream, are not true hydrological units, and they are difficult to delineate and explain the hydrological and ecological condition of the stream validly. To overcome this, our study defines the multi-scale nested watersheds based on the basic watershed units created by a digital elevation model for the purpose of more effective watershed management, and multi-scale analysis is adopted to explore the relationships between agricultural land use intensity and water quality, and further to identify watershed adaptive response units for every water quality parameter.

Beijing’s mountainous watersheds, providing 69.9% of its surface water resources, have played increasingly important roles in drinking water supply and headwater conservation considering the population increase and urban sprawl of Beijing. Moreover, land use changes in the Beijing mountainous areas have brought about many land related problems, such as water pollution, soil contamination and air pollution [23]. We had adopted emergy analysis with principal component analysis, regression analysis and cluster classification to investigate the characteristics and patterns of agricultural land use intensity of study areas in 2000, as the baseline of ecological monitoring and assessment [24]. However, the effects of the agricultural land use intensity on surface water quality have not been discussed. Therefore, the objective of this study, taking the Beijing mountainous area as a case, was to investigate the impacts of agricultural land use intensity on selected physical properties of surface water quality using multi-scale analysis for building a baseline database applicable to long-term monitoring.

## 2. Materials and Methods

### 2.1. Study Areas

**Figure 1:**
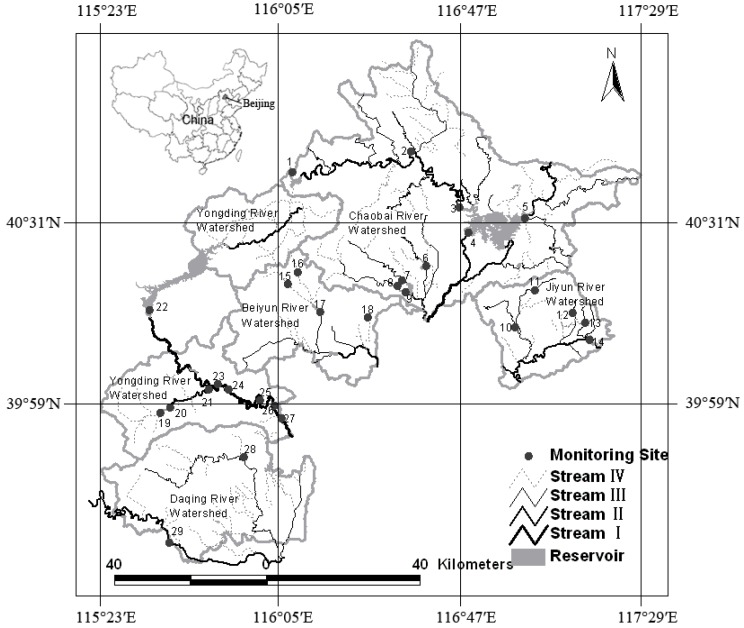
Study area and monitoring sites.

Beijing’s mountainous areas, with an area of 1.04 × 10^6^ ha, are located to the west, north and northeast of Beijing. The study areas comprise a total of five rivers, including the Yongding River, Chaobai River, Beiyun River, Jiyun River and Daqing River (Figure 1). Mean annual precipitation in the area is about 566 mm, about 60% of which falls in July and August. The annual average evaporation is about 1,761 mm. Annual average runoff was about 1.8 × 10^9^ m^3^, but this had decreased to 1.3 × 10^9^ m^3^ by the end of the last century as a result of climate and land use/land cover changes.

**Figure 2:**
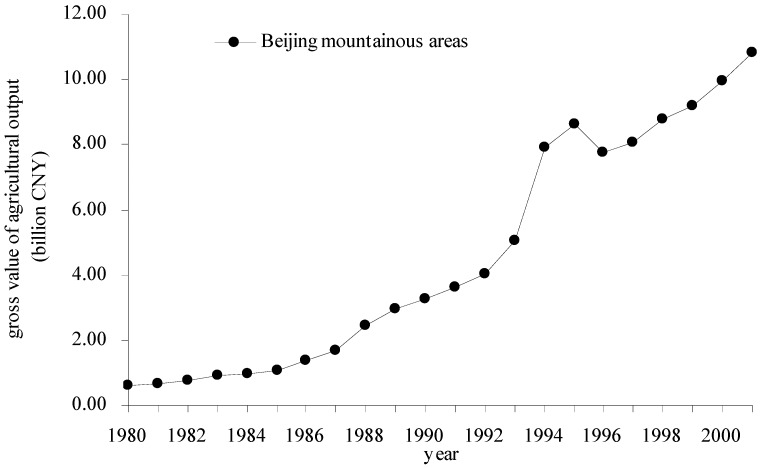
Gross agricultural output for the Beijing mountainous areas.

With the population increase and urban sprawl of Beijing, mountain agriculture has played increasingly important roles in areas such as services, the economy, ecological security and tourism. Figure 2 shows that the gross value of agricultural output in the study area increased quickly with the pressure for arable land resources in plain areas that have become non-agricultural land owing to city sprawl, particularly in the high development periods of the mid 1990s. The past studies suggested that the increase in agricultural output mainly depended on the input of a large amount of non-renewable resources, especially agricultural chemical fertilizers, pesticides and plastic film, according to the correlation analyses of agricultural inputs and outputs in 2000 [24]. The main non-renewable agricultural inputs have still increased in recent decade years, which has led to greatly increased environmental damage such as water pollution, soil contamination and air pollution caused by agriculture, especially high intensity industrialized agriculture.

### 2.2. Surface Water Quality Data

There are 27 monitoring sites in the study area. The monitoring Sites 1–8, 9–13, 14–17, 18–25 and 26–27 correspond to the Chaobai River, Juyun River, Beiyun River, Yongding River and Daqing River watersheds, respectively (Figure 1). The streams on which they are respectively located are listed in Table 1. Water samples were taken at these stations monthly from May to October 2000. Of these, June, July, August and November in 2000 were the rainy reason, and the rest was the dry season. A total of 162 samples were collected at the 27 sites of the Beijing mountainous areas. Water samples were analyzed in the laboratory for eight water quality characteristics, including nitrate-nitrogen (NO_3_-N), permanganate index (COD_Mn_), biochemical oxygen demand for five days (BOD_5_), total nitrogen (TN), total phosphorus (TP), total mercury (Hg), total cadmium (Cd) and total lead (Pb). Then the monthly average values of water quality characteristics for each site were derived, which were used for our statistical assessment.

**Table 1:** The streams and watersheds of the 27 monitoring sites.

Watersheds	Stream	Site
Chaobai River	Bai River	1–3
	Chao River	4
	Yanqi River	5
	Huaisha River	6
	Huaijiu River	7
	Huai River	8
Jiyun River	Cuo River	9
	Zhenluoying Rock River	10
	Huangsongyu Rock River	11
	Jiangjunguan Rock River	12
	Ju River	13
Beiyun River	Deshengkou Ditch	14
	Zhuishikou Ditch	15
	Dongsha River	16
	Qintun River	17
Yongding River	Qingshui River	18–20
	Yongding River	21–25
Daqing River	Dashi River	26
	Juma River	27

The surface water quality characteristics NO_3_-N and COD_Mn _have been considered as two of the four water pollutant load control indexes in China. Furthermore, the relationship between water quality characteristics was tested at the significance levels of *p *< 0.01 and 0.05, which showed that NO_3_-N had a high positive correlation with TN, TP, Hg, Cd and Pb, and COD_Mn_ had a positive correlation with Hg, Cd and Pb (Table 2).

Therefore, we focused on the two conventional water quality characteristics NO_3_-N and COD_Mn_, which not only can reduce the complexity of the study, but also is of great significance for water resources management.

**Table 2:** Bivariate correlation coefficients of water quality variables.

	NO_3_-N	COD_Mn_	BOD_5_	Hg	Cd	Pb	TN	TP
NO_3_-N	1							
COD_Mn_	0.607^ b^	1						
BOD_5_	0.655	–0.034	1					
Hg	0.809^ b^	0.857^ b^	–0.611	1				
Cd	0.917^ b^	0.640^ b^	–0.742	0.904^ b^	1			
Pb	0.917^ b^	0.640^ b^	–0.742	0.904^ b^	1.000^ b^	1		
TN	0.996^ a^	–0.829	0.587	0.282	0.107	0.107	1	
TP	0.999^ a^	–0.805	0.621	0.242	0.065	0.065	0.999^ a^	1

^a ^Significant at the 0.01 level. ^b ^Significant at the 0.05 level.

### 2.3. Multi-Scale Watershed Delineation

#### 2.3.1. Basic Watershed Units

**Figure 3:**
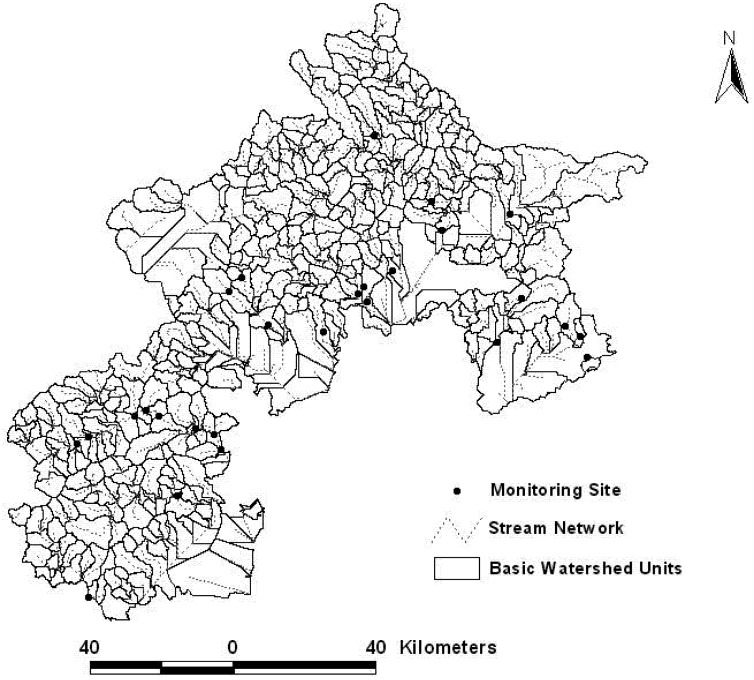
Basic watershed units delineated in the study area.

A watershed is the up-slope area contributing flow to a given location. Such a feature is also variously referred to as a catchment or basin, and comprises a part of a hierarchy in that any given watershed is generally part of a larger watershed [19]. A digital elevation model (DEM) with 30 m × 30 m grid resolution was adopted to create basic watershed units for the Beijing mountainous area using the hydrologic functions in the ArcView extensions. The minimum number of cells for a stream network in the hydrologic functions is very important for the watershed delineation. Many studies have shown that when the minimum number of cells was smaller, the extracted stream networks were denser, and the created basin areas were smaller. The stream network extracted by hydrologic functions should be similar to that existing in Nature, and each of the monitoring sites should be located at the pour point of different basic watershed units for the purpose of this study. Therefore, the thresholds of 15,000, 12,000, 10,000, 8,000 and 5,000 were chosen as the minimum number of cells for a stream network during the test of basic watershed unit creation. The results indicated that a few monitoring sites were located in the same basic watershed unit when the thresholds was bigger than 10,000, and the stream networks extracted were far denser than natural stream network when the threshold was less than 10,000. Ultimately, the thresholds of 10,000, *i*.*e*., 900 ha, which is far smaller than the average area of the Beijing mountain towns (9,285 ha), was determined as the minimum number of cells for a stream network to delineate basic watershed units (Figure 3).

#### 2.3.2. Delineating Multi-Scale Watersheds as Watershed Analysis Units

Multi-scale watersheds corresponding to the 27 monitoring sites were identified as watershed analysis units for further analysis according to the flow direction and rivers, respectively. Since some monitoring sites are located on the same streams, such as Sites 1–3 (Bai River), Sites 18–20 (Qingshui River) and Sites 21–25 (Yongding River), perhaps they are statistically highly correlated and, to some extent, all the upstream points contribute to the measurements of any monitoring point. This is statistically undesirable and would produce strongly biased results. To solve this problem, the independence of these sites’ data should be tested. A serial autocorrelation test was adopted to analyze the possible correlation between neighboring sites. The test results showed that these upstream monitoring sites had little contribution to their nearest downstream sites for NO_3_-N and COD_Mn_ water quality data in 2000. Therefore, the monitoring samples can be considered independent for further statistical analysis, and the watersheds contributing flow to these sites cannot be included in the their nearest downstream sites’ watersheds when delineating the watershed analysis units.

**Figure 4:**
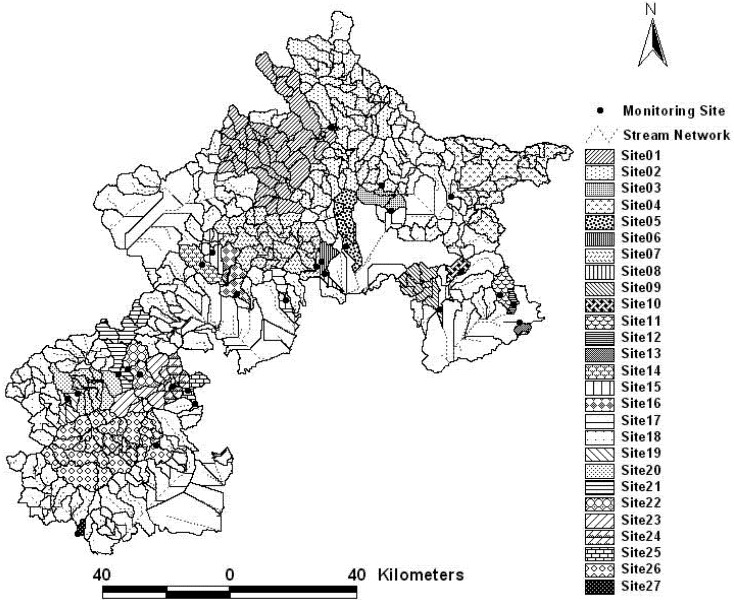
The whole watersheds for the 27 monitoring sites.

**Figure 5:**
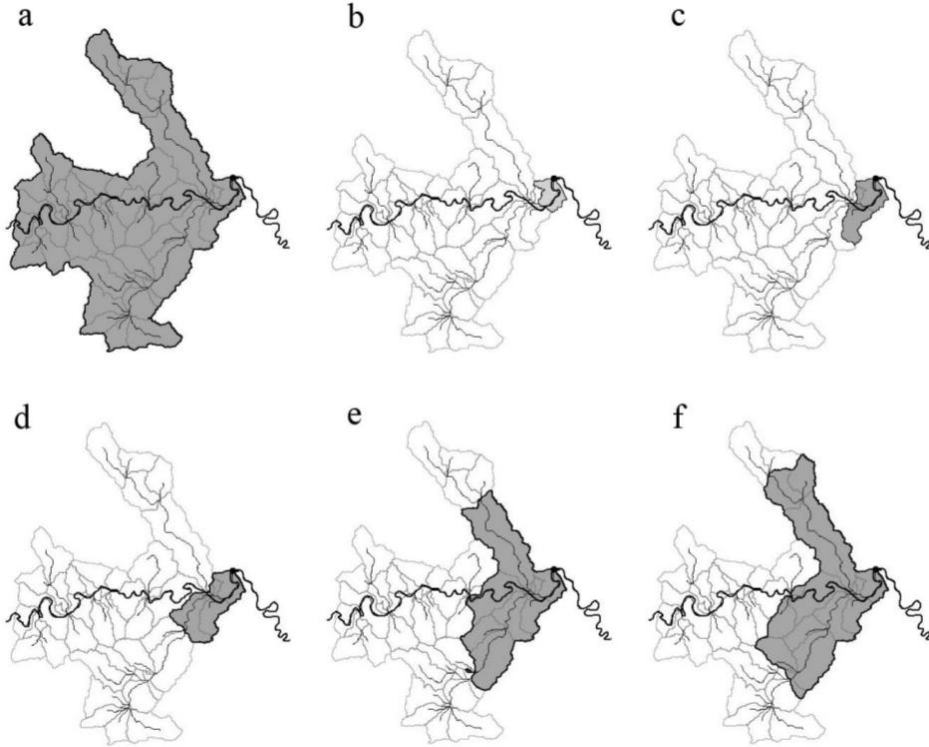
Illustration of how the multi-scale watersheds were defined: (**a**) The whole watershed for Site 1. (**b**) Definition of the Zone 1. (**c**) Definition of the Zone 2. (**d**) Definition of the Zone 3. (**e**) Definition of the Zone 4. (**f**) Definition of the Zone 5.

Firstly, the basic watershed units contributing flow to every monitoring site were delineated as the whole watershed of every monitoring site, which did not have a nest relation according to the result of serial autocorrelation test mentioned above (Figure 4). Table 3 shows the number of towns covered by the whole watershed for every monitoring site. Subsequently, multi-scale watersheds were determined in the whole watershed area of every monitoring site. The basic watershed units directly contributing flow to every monitoring site were considered as the first order watershed (Zone 1). The Zone 1 and the basic watershed units directly contributing flow to the Zone 1 were together defined as second order watershed (Zone 2). By analogy, the next order watersheds were derived and named in order third order watershed (Zone 3), fourth order watershed (Zone 4) and fifth order watershed (Zone 5), *etc. *Figure 5 illustrates the process of delineating multi-scale watersheds for Site 1. Table 3 also lists the number of scales for every monitoring site.

**Table 3:** The number of scales and towns be covered for 27 monitoring sites.

Site	Towns	Scale	Site	Towns	Scale
1	6	>10	14	3	1
2	7	>10	15	2	3
3	2	2	16	4	4
4	6	7	17	1	2
5	4	3	18	1	7
6	2	1	19	2	3
7	5	10	20	2	8
8	3	1	21	2	4
9	4	5	22	2	2
10	2	1	23	4	6
11	4	1	24	4	3
12	3	1	25	2	2
13	2	1	26	8	>10
			27	1	1

### 2.4. Agricultural Land Use Intensity for Watershed

With more attention being paid to land use and land cover change, an approach to assess agricultural land use intensity including agricultural input and output intensity on a general basis has been developed in our previous work [24]. The measurement and assessment of agricultural land use intensity was preformed at municipal/town level because the agricultural inputs and outputs information derived from census data are aggregated and officially reported at this level, and this level also is the smallest administrative unit as planning and management purpose in China. Four indices of agricultural input intensity and six of output intensity have been derived with principal component analysis at this level for the Beijing mountainous areas using the amount of emergy of each agricultural input and output derived from agricultural census data [24]. Their eigenvectors are given in Table 4 and Table 5. The several indices reflecting agricultural input and output intensity were dimensionless, and the meanings of these indices according to their eigenvectors are shown in Table 6. The higher the index value, the greater the agricultural input or output intensity.

**Table 4:** Eigenvectors of the input intensity [24].

Components	IPC_1_	IPC_2_	IPC_3_	IPC_4_
Sunlight	0.990	−0.075	−0.083	0.018
Rain, chemical energy	0.983	−0.056	−0.086	0.070
Rain, geopotencial energy	0.932	−0.163	−0.159	−0.096
Earth cycle	0.991	−0.071	−0.086	0.028
Wind, kinetic energy	0.991	−0.071	−0.086	0.028
Soil loss	0.991	−0.071	−0.086	0.028
Agricultural electricity	−0.252	0.364	0.527	0.156
Nitrogen fertilizer	−0.102	0.792	0.165	0.448
Phosphorus fertilizer	−0.062	0.852	0.011	0.305
Potash fertilizer	−0.103	0.890	0.113	0.139
Compound fertilizer	−0.119	0.809	0.270	0.184
Pesticides	0.005	0.109	0.787	−0.103
Agricultural plastic mulch	−0.165	−0.024	0.673	0.180
Machinery power	0.003	0.619	0.348	−0.085
Human labor	0.140	0.344	0.620	0.494
Livestock labor	0.135	0.020	−0.034	0.875
Organic manure	−0.088	0.471	0.268	0.648
Seed	−0.090	0.761	0.234	0.425

**Table 5:** Eigenvectors of the output intensity [24].

Components	OPC_1_	OPC_2_	OPC_3_	OPC_4_	OPC_5_	OPC_6_
Grain crops	0.569	0.423	0.258	0.186	0.055	0.045
Oil crops	−0.021	−0.003	−0.050	0.911	0.056	0.046
Vegetables	0.761	0.136	−0.105	−0.156	−0.028	−0.092
Fruits	−0.077	−0.088	0.867	−0.131	0.088	0.064
Pork	0.682	0.228	0.226	0.325	0.187	−0.033
Beef	0.240	0.792	−0.061	0.063	−0.139	0.096
Mutton	−0.053	0.109	0.069	0.050	0.021	0.967
Fowl	0.359	0.094	0.548	0.389	−0.175	−0.002
Milks	0.097	0.855	−0.026	−0.071	0.146	0.008
Eggs	0.815	0.111	−0.038	0.003	0.055	−0.124
Forest logging	0.062	0.036	0.026	0.048	0.968	0.020
Fish	0.781	−0.237	−0.077	−0.120	−0.101	0.225

**Table 6:** The indices of agricultural input and output intensity.

	Indices at town level	Meanings	Indices at watershed level
Agricultural input intensity	IPC_1_	Natural factors, including soil loss, rainfall and sunlight	WI-IPC_1_
	IPC_2_	Chemical fertilizer, seed and mechanized power	WI-IPC_2_
	IPC_3_	Pesticides, agricultural plastic mulch, human labor and agricultural electricity	WI-IPC_3_
	IPC_4_	Organic manure	WI-IPC_4_
Agricultural output intensity	OPC_1_	Eggs, vegetables, pork and grain crops	WI-OPC_1_
	OPC_2_	Milks and beef	WI-OPC_2_
	OPC_3_	Fruits and fowl	WI-OPC_3_
	OPC_4_	Oil crops	WI-OPC_4_
	OPC_5_	Forest cut	WI-OPC_5_
	OPC_6_	Sheep	WI-OPC_6_

For watershed-scale analysis, the indices of agricultural input and output intensity should be translated from the municipal/town level to the watershed level. Because there is a spatial incompatibility between the watershed analysis unit and the municipal/town unit, the weighted values with the percentage of town’s agricultural land area in the watershed analysis unit were used as the weights to calculate the agricultural land use intensity indices for watershed analysis unit, which is as follows:

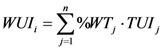
(1)
where *WUI_i_* are the agricultural input and output indices for the watershed analysis unit *i*, %*WT_j_* is the percentage of town *j*’s agricultural land area in a watershed analysis unit *i*. *TUI_j_* is the agricultural input and output indices for town *j*. Therefore, the indices for watershed analysis unit *i* were the weighted values with agricultural land area percentage used to reflect agricultural input and output intensity at every watershed scale.

### 2.5. Agricultural Land Use Intensity and Water Quality Linkage

The question of a relationship between agricultural land use intensity and water quality was examined at various scales by applying multiple regression techniques considering nutrient concentrations as dependent variables and the agricultural land use intensity as explanatory variables. The functional form of the relationship for each of these scales is as follows:

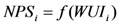
(2)
where *NPS_i_* is nutrient concentration for monitoring site in question in watershed analysis unit *i*, *WUI_i_* is equal to the indices of agricultural input and output intensity for watershed analysis unit *i*.

In previous studies, the concentration of nutrients over an area can be described in the form of an exponential model or a linear model considering the proportions of land use/land cover as explanatory variables. Delivery of non-point source pollutants from discrete upstream contributing zones to a particular downstream point is a multi-step, often episodic, process [25]. A first order rate equation can be used for modeling nutrient attenuation in flow through various land uses to the nearest stream [25]. Since agricultural land use intensity has been one of the most significant forms of land cover modification, the exponential model was chosen for this research to explore the relationship between agricultural land use intensity and water quality, and to recognize how agricultural land use intensity affects water quality at various watershed scales. Stepwise multiple regression analysis was performed using log transformed dependent variables to reduce the asymmetric distribution of the data. The numbers of scales for 27 monitoring sites were different, thus, the used monitoring sites and sample number at various scales were different in the multi-scale regression analyses (Table 7). Because the sample numbers were too small to make valuable regression analysis above the scale of Zone 5, the multi-scale regression analyses were carried out with SPSS 13.0 at the scales from Zone 1 to Zone 5. Based on this statistical analysis, watershed adaptive response units for each water quality variable can also be identified.

**Table 7:** The monitoring sites at various scale level in the multi-scale analyses.

Scale	Sites	Number
Zone 1	1, 2, 3, 4, 5, 6, 7, 8, 9, 10, 11, 12, 13, 14, 15, 16, 17, 18, 19, 20, 21, 22, 23, 24, 25, 26, 27	27
Zone 2	1, 2, 3, 4, 5, 7, 9, 15, 16, 19, 20, 21, 22, 23, 24, 25, 26	17
Zone 3	1, 2, 4, 5, 7, 9, 15, 16, 19, 20, 21, 23, 24, 26	14
Zone 4	1, 2, 4, 7, 9, 16, 20, 21, 23, 26	10
Zone 5	1, 2, 4, 7, 9, 20, 23, 26	8
Zone 6	1, 2, 4, 7, 20, 23, 26	7
Zone 7	1, 2, 4, 7, 20, 26	6
>Zone 7		<5

## 3. Results

### 3.1. Water Quality Assessment

Table 8 shows the 2000 yearly mean concentration of NO_3_-N and COD_Mn_. The concentration of NO_3_-N in the samples ranged from 0.46 to 12 mg/L, while COD_Mn_ ranged from 1 to 7.4 mg/L. Only the concentration of NO_3_-N for Site 12 exceeded to 10 mg/L, which is the Chinese surface drinking water standard limit. Different from NO_3_-N, there are five types of surface water environmental quality standard for COD_Mn_. Since type IV and V cannot be acceptable for drinking water, the type III recommended value of 6 mg/L is considered as the surface drinking water standard limit for COD_Mn_. According to this standard limit of 6 mg/L, Site 12 was faced with organic contaminant and reducible inorganic substance drinking water pollution. Since Sites 10, 11, 17 and 22 were close to the standard limit of 6 mg/L, especially Site 10, they also had a pollution risk caused by COD_Mn_.

**Table 8:** The concentration of NO_3_-N and COD_Mn_ in stream water of 27 monitoring sites.

Monitoring site	NO_3_-N			COD_Mn_		
	NO_3_-N (mg/L)	Standard limit	Type	COD_Mn_ (mg/L)	Standard limit	Type
1	1.6	10	Not exceeding	2.4	4	II
2	1.26	10	Not exceeding	2.2	4	II
3	0.79	10	Not exceeding	2.3	4	II
4	3.14	10	Not exceeding	2.1	4	II
5	0.46	10	Not exceeding	3.2	4	II
6	1.67	10	Not exceeding	1.9	2	I
7	2.69	10	Not exceeding	1.5	2	I
8	0.66	10	Not exceeding	2.5	4	II
9	1.95	10	Not exceeding	1.5	2	I
10	3.4	10	Not exceeding	6	6	III *
11	2.57	10	Not exceeding	4.4	6	III *
12	12	10	Exceeding *	7.4	10	IV **
13	1.09	10	Not exceeding	2.6	4	II
14	0.86	10	Not exceeding	1.4	2	I
15	1.06	10	Not exceeding	1.5	2	I
16	0.37	10	Not exceeding	2.7	4	II
17	0.18	10	Not exceeding	4.9	6	III *
18	1.68	10	Not exceeding	1.4	2	I
19	1.72	10	Not exceeding	3.2	4	II
20	1.78	10	Not exceeding	1.3	2	I
21	1.88	10	Not exceeding	2.1	4	II
22	1.6	10	Not exceeding	4.4	6	III *
23	1.33	10	Not exceeding	4	4	II
24	1.51	10	Not exceeding	3.9	4	II
25	1.51	10	Not exceeding	4	4	II
26	3.51	10	Not exceeding	1	2	I
27	1.76	10	Not exceeding	1.6	2	I

### 3.2. Linkage Model Results at Multiple Scales

The regression models of NO_3_-N and COD_Mn_ at the various watershed scales are shown in Table 9 and Table 10, respectively. The log-transformed NO_3_-N exhibited a relationship with WI-IPC_1_ and WI-IPC_2_ at the scale of Zone 1, while no statistically significant relationships were found between agricultural land use intensity and nitrate level at the other watershed scales. The regression equation in the Zone 1 model, with the value 0.374 of R^2^ and the level 0.004 of statistical significance, is as follows:

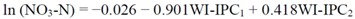
(3)


Although the coefficients of determination R^2^ is relatively weak, the model is statistically significant. The model suggests that natural factors act as sinks, and as the input intensity of natural factors including rainfall and sunlight inside the contributing zone (Zone 1) increases, NO_3_-N levels downstream decrease. In addition, several artificial inputs, especially chemical fertilizer input, are identified as the second largest contributors of NO_3_-N, and as chemical fertilizer input intensity within the contributing zone (Zone 1) increases, NO_3_-N levels downstream also increase.

**Table 9:** Multiple regression model of NO_3_-N.

Scales	Variable in equation	Standardized Beta	R^2^	Sig.	Number of samples
Zone1	WI-IPC_1_	−0.469	0.374	0.004	27
	WI-IPC_2_	0.412			
Zone2	no variables were entered				17
Zone3	no variables were entered				14
Zone4	no variables were entered				10
Zone5	no variables were entered				8

**Table 10:** Multiple regression model of COD_Mn_.

Scales	Variable in equation	Standardized Beta	R^2^	Sig.	Number of samples
Zone1	no variables were entered				27
Zone2	no variables were entered				17
Zone3	no variables were entered				14
Zone4	no variables were entered				10
Zone5	WI-IPC_3_	0.527	0.452	0.001	8
	WI-IPC_4_	0.085			

For the water quality index COD_Mn_, no variables were entered in the stepwise regression analysis for the scales from Zone 1 to Zone 4, while the most important explanatory variables were the WI-IPC_3_ and WI-IPC_4_ at the watershed scales of Zone 5. The regression equation between agricultural land use intensity and permanganate index in the Zone 5 model, with the value 0.452 of R^2^ and the level 0.001 of statistical significance, is as follows:

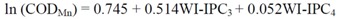
(4)


In the regression model of permanganate index, the two input intensities of pesticides and agricultural plastic mulch and organic manure inside the contributing zone (Zone 5) both have the positive impact on the permanganate level downstream. Therefore, the input of pesticides and agricultural plastic mulch is considered as the larger contributor than the organic manure input.

## 4. Discussion and Conclusions

### 4.1. Agricultural Input Intensity and Surface Water Quality Risk

Land use/land cover management, particularly high-input agriculture, is considered to be an important source of pollution export from catchments and frequently has been identified as a major contributor of surface water pollution [26]. The above results and analysis provide insight into the linkages between agricultural land use intensity and regional surface water quality. For the Beijing mountainous study area, several groups of agricultural input affecting surface water quality were identified during the year 2000. The results indicated that the explanatory variables behind the various water quality indexes were quite different at the respective significant watershed scales. The view that nitrate may be a useful general indicator of intensive land use was supported by previous work by Hunt *et al* [27]. As in Hunt [27], nitrate in particular can be considered as a useful indicator of intensive natural factors and agricultural chemical fertilizer input at the significant watershed scale in the Beijing mountainous areas. The input intensity of pesticide and agricultural plastic mulch in the Beijing mountainous areas watersheds has increased drastically between 1984 and 2000, which resulted in the permanganate index pollution risk. Several studies have indicated that the proportion of vegetable-planted land exhibited a positive correlation with permanganate index [28]. For the Beijing mountainous areas, vegetable outputs depended principally on the abundant inputs of pesticide and agricultural plastic mulch in 2000, according to our previous research [24].

### 4.2. Watershed Adaptive Response Unit

The significant scales at which there were statistically significant relationships between agricultural land use intensity and each water quality variable were identified on the basis of the multi-scale regression analysis, which were considered as the watershed adaptive response units for each water quality variable. Thus, the first order watershed (Zone 1) of 27 monitoring points was the adaptive response unit for nitrate-nitrogen, while the fifth order watershed (Zone 5) was the adaptive response unit for the permanganate index.

In the Beijing mountainous study area, watershed adaptive response units differ with the water quality variables being assessed, which are related with transformation regularity of nitrate-nitrogen and permanganate index. After the use of nitrate fertilizer that is the source of nitrate-nitrogen on agricultural fields, nitrate-nitrogen formed by nitrification is either absorbed and utilized by plants or transformed into gaseous nitrogen through denitrification under reducing conditions. Therefore, only the agricultural inputs inside the first order watershed zones can make a significant contribution to the concentration of nitrate-nitrogen at the pour-point, while that inside other order watershed zones has little influence on the nitrate-nitrogen level at the pour-point with the action of long distance transport.

Compared to nitrate-nitrogen, permanganate index contamination is relatively steady. As the main source, the transformation time of agricultural plastic mulch and pesticides is relatively long. It could not make a significant contribution to the concentration of permanganate index until it accumulates. Therefore, the smaller area basins, such as first order watershed to the fourth order watershed, hardly respond to permanganate index as a contributing zone.

The definition of watershed adaptive response unit based on the basic watershed units, actually a contributing zone, is very meaningful for the purpose of more effective watershed management. It is important to address the fine-scale management issues relate to watershed adaptive response units for every water quality parameter. The adjustment of agricultural input structure and intensity may be carried out inside the individual watershed adaptive response units.

### 4.3. Modeling

The multiple linear regression model performed using log transformed dependent variables, which was adopted in many previous studies to explore the relationship between land use and stream water, can also provide insight into the linkages between agricultural land use intensity and stream water quality at multiple watershed scales. The statistical models in this study are valuable in examining the relative sensitivity of water quality indexes to alterations in agricultural land use intensity inside the various contributing zones when coupled with expert knowledge. The modeling results can also further help to identify the cause-and-effect relationships between agricultural input intensity and stream water quality inside the watershed adaptive response units, which are important in the management of water quality. The modeling, although statistically significant, showed the relatively weak coefficients of determination. It may be that the spatial incompatibility between the watershed spatial unit and the municipal/town unit was actually existed, or that other potential factors influencing stream water quality variable were not included in the analysis. All of these are worthy of further research.

Although multiple linear regression models are an effective approach for identifying significant agricultural input intensity affecting water quality and explaining the relationship between agricultural land use intensity and stream water quality, they do not appear to quantitatively estimate contribution of respective agricultural land use intensity on the water quality because they are only based on the existence of statistical significance in the analysis data, rather than any mechanistic relationships between sources and receptors. Our future research will focus on understanding the exact mechanisms of the effect of agricultural land use intensity on stream water quality by adopting an alternative “sources-receptors model” based on the mass balance approach.
